# Multiple SARS-CoV-2 Variants Exhibit Variable Target Cell Infectivity and Ability to Evade Antibody Neutralization

**DOI:** 10.3389/fimmu.2022.836232

**Published:** 2022-03-16

**Authors:** Haijun Tang, Long Gao, Zhao Wu, Fang Meng, Xin Zhao, Yun Shao, Guocun Hou, Xiaohong Du, F. Xiao-Feng Qin

**Affiliations:** ^1^ Institute of Systems Medicine, Chinese Academy of Medical Sciences & Peking Union Medical College, Beijing, China; ^2^ Suzhou Institute of Systems Medicine, Suzhou, China; ^3^ Department of Nephrology, Suzhou Science and Technology Town Hospital, Suzhou, China; ^4^ Institute of Clinical Medicine Research, Suzhou Science and Technology Town Hospital, Suzhou, China

**Keywords:** SARS-CoV-2 variants, infectivity, CoronaVac vaccine, neutralizing antibody, BBIBP-CorV vaccine

## Abstract

The continuous emergence of SARS-coronavirus 2 (SARS-CoV-2) variants, especially the variants of concern (VOC), exacerbated the impact of the coronavirus disease 2019 (COVID-19) pandemic. As the key of viral entry into host cells, the spike (S) protein is the major target of therapeutic monoclonal antibodies (mAbs) and polyclonal antibodies elicited by infection or vaccination. However, the mutations of S protein in variants may change the infectivity and antigenicity of SARS-CoV-2, leading to the immune escape from those neutralizing antibodies. To characterize the mutations of S protein in newly emerging variants, the proteolytic property and binding affinity with receptor were assessed, and the vesicular stomatitis virus (VSV)-based pseudovirus system was used to assess the infectivity and immune escape. We found that some SARS-CoV-2 variants have changed significantly in viral infectivity; especially, B.1.617.2 is more likely to infect less susceptible cells than D614G, and the virus infection process can be completed in a shorter time. In addition, neutralizing mAbs and vaccinated sera partially or completely failed to inhibit host cell entry mediated by the S protein of certain SARS-CoV-2 variants. However, SARS-CoV-2 variant S protein-mediated viral infection can still be blocked by protease inhibitors and endocytosis inhibitors. This work provides a deeper understanding of the rise and evolution of SARS-CoV-2 variants and their immune evasion.

## Introduction

The coronavirus disease 2019 (COVID-19) pandemic is threatening human health worldwide. As of November 23, 2021, SARS-coronavirus 2 (SARS-CoV-2) has infected over 256 million people and has caused more than 5 million deaths (https://covid19.who.int). SARS-CoV-2 uses its spike (S) protein to bind host receptor angiotensin-converting enzyme 2 (ACE2) and mediate virus entry into host cells (1–5). Virus entry requires the target cell protease to activate the S protein, which triggers the cleavage between S1 and S2, activates the cleavage of the S2 site, and then mediates the fusion of the virus with the cell membrane (6–8). SARS-CoV-2 entry can be disrupted by protease inhibitors or endocytosis inhibitors (6, 8). The therapeutic applicability of these drugs for COVID-19 treatment is being evaluated within clinical trials.

The number of COVID-19 confirmed cases continues to grow rapidly, and the prospect of ending its pandemic depend on effective treatment and prevention measures. SARS-CoV-2 S protein, especially receptor-binding domain (RBD), is the main target of neutralizing monoclonal antibodies (mAbs). At present, neutralizing mAbs have shown a good therapeutic efficacy on infected individuals (9). Some antibodies have been shown to reduce viral load, relieve COVID-19 associated symptoms, and reduce hospitalization rate (10, 11). In addition, vaccines play a key role in the prevention and control of the COVID-19 epidemic in various regions of the world. At present, SARS-CoV-2 vaccines, including mRNA vaccines, adenovirus-based vaccines, protein subunit vaccines, and inactivated vaccines, all show encouragingly good clinical efficacy (12, 13). CoronaVac, an inactivated vaccine developed by Sinovac, has been demonstrated to be more than 50% effective against symptomatic infections and can reduce the risk of serious diseases (14). Similarly, another inactivated vaccine, BBIBP-CorV, developed by Sinopharm, has been confirmed to render 78.1% protection efficacy against COVID-19 symptomatic infections and reduce the hospitalization rate of patients (15).

Although the genome of SARS-CoV-2 remains relatively stable, it still has a high mutation rate during virus transmission (16–18). With the extension of the SARS-CoV-2 pandemic, a wide variety of variants have been produced in the host. In particular, SARS-CoV-2 S protein G614 has replaced the original D614 and has become the main popular SARS-CoV-2 strain (19–21). Recently, several new SARS-CoV-2 variants have appeared, which seem to be more infectious in the population. The variants make their antigenicity different from the original strain, which can reduce or even invalidate the protective efficacy of current antibodies and vaccines (18). Among the variants of concern (VOC), the B.1.351 and P.1 strains have attracted wide attention because of their extensive mutations and the ability to escape from neutralizing antibodies (22, 23). Several studies have shown that the power of neutralizing antibodies and vaccinated sera are substantially reduced in neutralization against B.1.351 and P.1 lineage variants (16, 24, 25). Moreover, due to enhanced transmission and immune evasion ability, B.1.617.2 has spread widely on a global scale, causing widespread concern (17, 26, 27). As widely employed SARS-CoV-2 vaccines in China, CoronaVac and BBIBP-CorV need to be tested for their protection ability against concerning variants.

In the work reported here, using a vesicular stomatitis virus (VSV)-based pseudovirus system, we have studied the infectivity, antigenicity, and drug inhibition characters of the major circulating SARS-CoV-2 variants. We show that some SARS-CoV-2 variants have changed significantly in viral infectivity. In addition, the antigenicity of some variants has also changed, resulting in a significant reduction in the neutralizing activity of some mAbs and vaccine sera. However, the entry of all variants into mammalian cells is effectively blocked by protease inhibitors and endocytosis inhibitors.

## Results

### Construction of Pseudoviruses With the Major Circulating SARS-CoV-2 Variants

**Figure 1:**
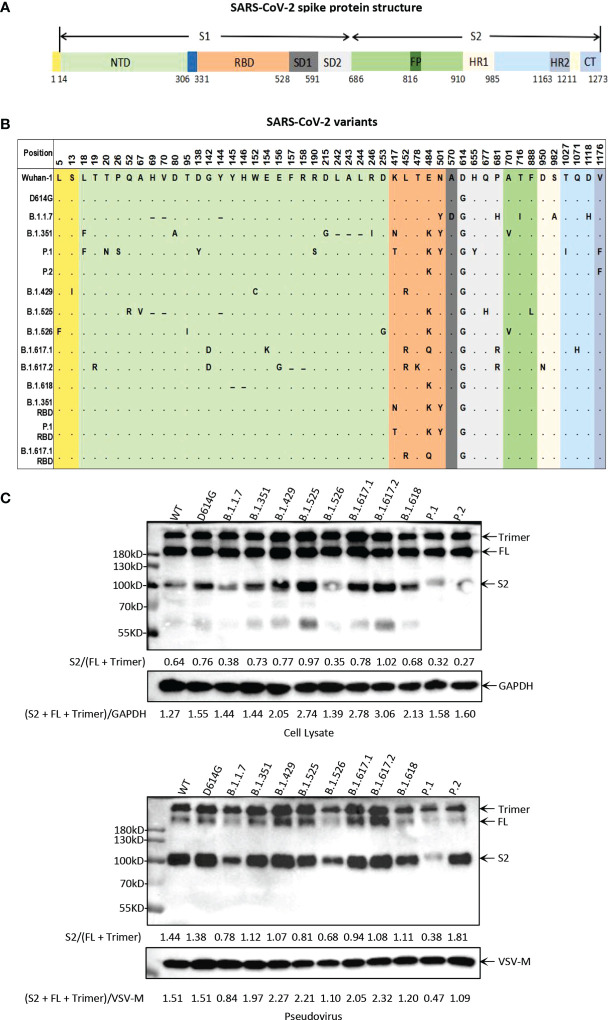
Illustration of the major circulating SARS-CoV-2 variants. **(A)** Schematic diagram of SARS-CoV-2 spike protein structure. NTD, N-terminal domain; RBD, receptor-binding domain; SD, subdomain; FP, fusion peptide; HR1, heptad repeat 1; HR2, heptad repeat 2; CT, cytoplasmic domain. **(B)** Mutation site at the amino acid level in the viral spike protein-coding region identified in SARS-CoV-2 variants. All of the pseudoviruses in this study were generated in the background of Wuhan-1 strain. In the mutation map, a dot (∙) indicates the same amino acid in that position as Wuhan-1 strain, and a dash (-) indicates a deletion. **(C)** Analysis of SARS-CoV-2 variants S protein expression and particle incorporation by Western blotting using a monoclonal antibody directed against SARS-CoV-2 S2 subunit. GAPDH (cell lysates) and VSV-M (particles) served as loading controls. The full-length S protein band is about 180 kDa, the S2 protein band is about 90 kDa, and the bands above 180 kDa represent trimeric S protein. Shown are representative blots from three experiments.

To study the biological characteristics of the major circulating SARS-CoV-2 variants, we generated a total of 14 pseudoviruses, including 11 main variants (D614G, B.1.1.7, B.1.351, P.1, P.2, B.1.429, B.1.525, B.1.526, B.1.617.1, B.1.617.2, and B.1.618) and 3 mutants with only RBD mutations (B.1.351 RBD, P.1 RBD, and B.1.617.1 RBD) (**Figures 1A, B** and **Figure S1**). In this study, all pseudoviruses were generated in the background of the Wuhan-1 virus strain, and D614G was used as the reference pseudovirus for the analysis of all experiments. We first analyzed the proteolytic process of the 11 main variant S proteins by Western blotting (**Figure 1C**). SARS-CoV-2 S protein mainly contains three bands, with the 180-kDa band reflecting the full-length S protein, the 90 kDa band reflecting the cleavage of S protein (S2 subunit), and the bands above 180 kDa representing trimeric S protein. The expression of full-length S protein can be detected in all variant strains, while the intensity of the S2 band was variable. The expression of S2 bands of B.1.1.7, P.1, and B.1.526 was weaker than that of D614G. But the expression of the S2 band of B.1.617.2 was stronger than that of D614G. Moreover, pseudoviruses with SARS-CoV-2 variant S protein were also analyzed for S protein incorporation and processing by Western blotting (**Figure 1C**). For all SARS-CoV-2 variants, the full-length S protein was cleaved into S2 protein to different extents. Similar to the expression of S protein in cell lysate, the intensity of S2 bands of B.1.1.7, P.1 and B.1.526 was weaker than that of D614G.

### Altered Infectivity of the Major Circulating SARS-CoV-2 Variants

**Figure 2:**
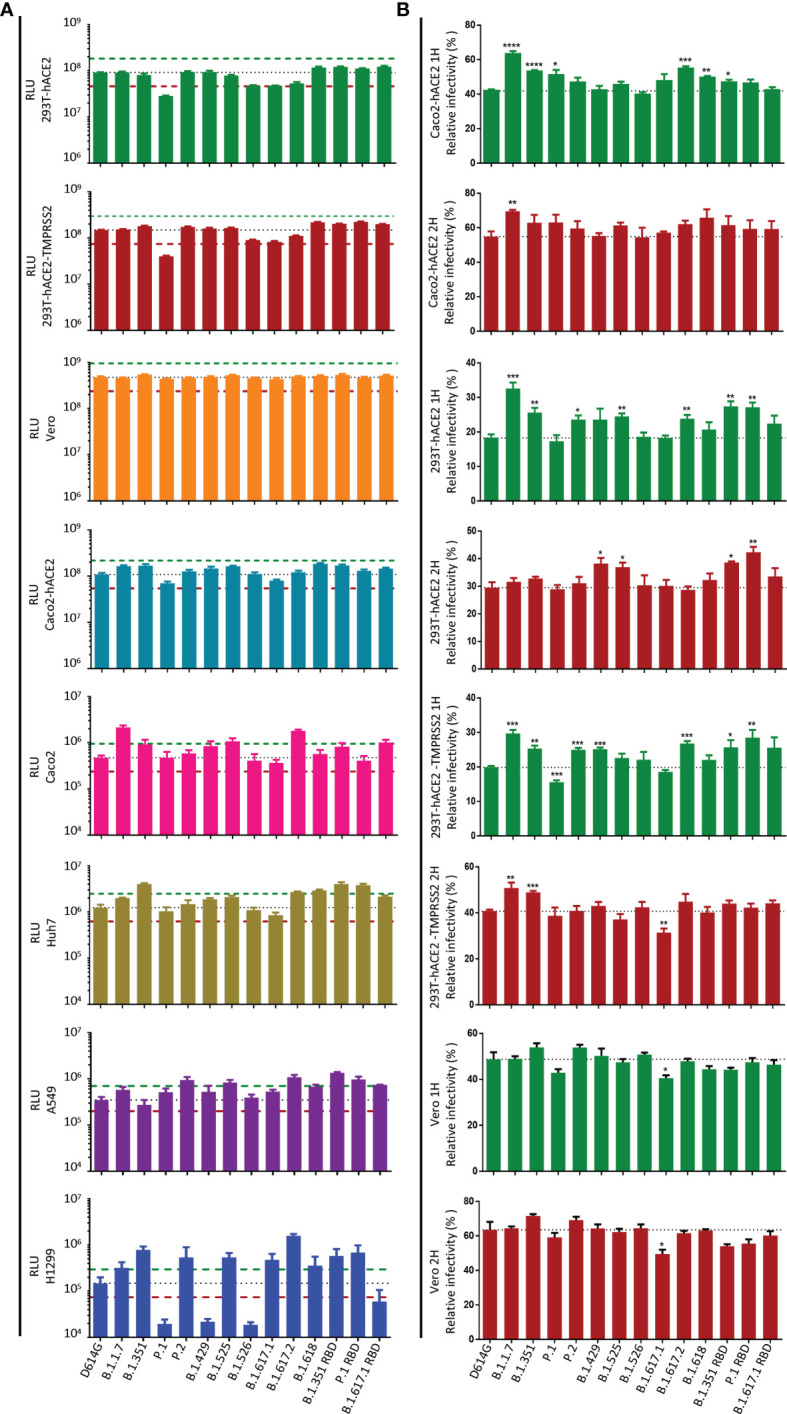
Infectivity analysis of SARS-CoV-2 variants in mammalian cell lines. **(A)** Entry of SARS-CoV-2 variants S pseudoviruses in mammalian cells. Mammalian cells were inoculated with pseudoviruses harboring SARS-CoV-2 variants S protein. At 24 h postinoculation, luciferase activity in cell lysates was measured to detect infection efficiency. The infectivity of the D614G variant was used as a control. The dashed lines indicate the threshold value of a 2-fold difference in infectivity. **(B)** SARS-CoV-2 variants S pseudoviruses were incubated with Caco2-hACE2, 293T-hACE2, 293T-hACE2-TMPRSS2, and Vero cells, then the unbound virus was removed by washing at the specified time point (1 or 2 h), and the unwashed cells were used as the control. At 24 h, luciferase activity in cell lysates was measured to detect infection efficiency. The infection efficiency of the virus was calculated by dividing the relative luminescence unit (RLU) value at each time point by the average RLU value of the respective virus at 24 h. A p-value less than 0.05 was defined as statistically significant (p < 0.05 [*], p < 0.01 [**], p < 0.001 [***], p < 0.0001 [****]). Experiments were done in 4 replicates and repeated at least twice. One representative is shown with error bars indicating SEM.

Next, we investigated the potential infection-related effects of pseudoviruses with variant S protein in different cells, where a difference by 2-fold in relative luminescence unit (RLU) value compared with the D614G strain was considered to be significant (**Figure 2A**). In the present study, the infectivity of the major circulating variants had no significant difference in 293T-hACE2, 293T-hACE2-TMPRSS2, Caco2-hACE2, and Vero cells, except that the infectivity of the P.1 variant was slightly reduced in 293T-hACE2 and 293T-hACE2-TMPRSS2 cells. On the other hand, in Caco2 cells, the infectivity of B.1.1.7, B.1.525, B.1.617.2, and B.1.617.1 RBD was significantly higher than that of D614G. In Huh7 cells, the infectivity of B.1.351, B.1.617.2, B.1.618, B.1.351 RBD, and P.1 RBD was higher than that of D614G. In addition, in A549 cells, the infectivity of P.2, B.1.525, B.1.617.2, B.1.351 RBD, P.1 RBD, and B.1.617.1 RBD was higher than that of D614G. Notably, in H1299 cells, the infectivity of B.1.1.7, B.1.351, P.2, B.1.525, B.1.617.1, B.1.617.2, B.1.618, B.1.351 RBD, and P.1 RBD was significantly enhanced, but the infectivity of P.1, B.1.429, B.1.526, and B.1.617.1 RBD was decreased. Interestingly, mutations outside the SARS-CoV-2 RBD may also affect viral infection. In A549 cells, B.1.351 RBD was more infective than B.1.351. Similarly, in 293T-hACE2, 293T-hACE2-TMPRSS2, Huh7, and H1299 cells, the infectivity of P.1 RBD was higher than that of P.1. In addition, B.1.617.1 RBD was more infective than B.1.617.1 in Caco2 cells but decreased in H1299 cells.

Zhang et al. (28) showed that pseudovirus carrying the delta variant S protein infects target cells faster than D614G in the early stage, which may be the main reason for its enhanced transmissibility. To test the ability of different variants to infect target cells in the early stage, we infected target cells with pseudoviruses carrying variant S protein and removed the unbound virus by washing at the specified time point (1 or 2 h), and the unwashed cells were used as the control. At 24 h post-infection, the infection efficiency of variants was detected by measuring the luciferase activity (**Figure 2B** and **Figure S2**). We found that in the early stage of virus infection, the infection efficiency of B.1.1.7, B.1.351, and B.1.617.2 in Caco2-hACE2, 293T-hACE2, and 293T-hACE2-TMPRSS2 cells was higher than that of the reference D614G strain. Compared with D614G, the infection efficiency of P.2, B.1.429, B.1.525, B.1.618, B.1.351 RBD, and P.1 RBD may be enhanced in some cells. However, all SARS-CoV-2 variants exhibited similar infectivity in Vero cells in the early stage.

### Binding of SARS-CoV-2 Variant S to Recombinant hACE2

**Figure 3:**
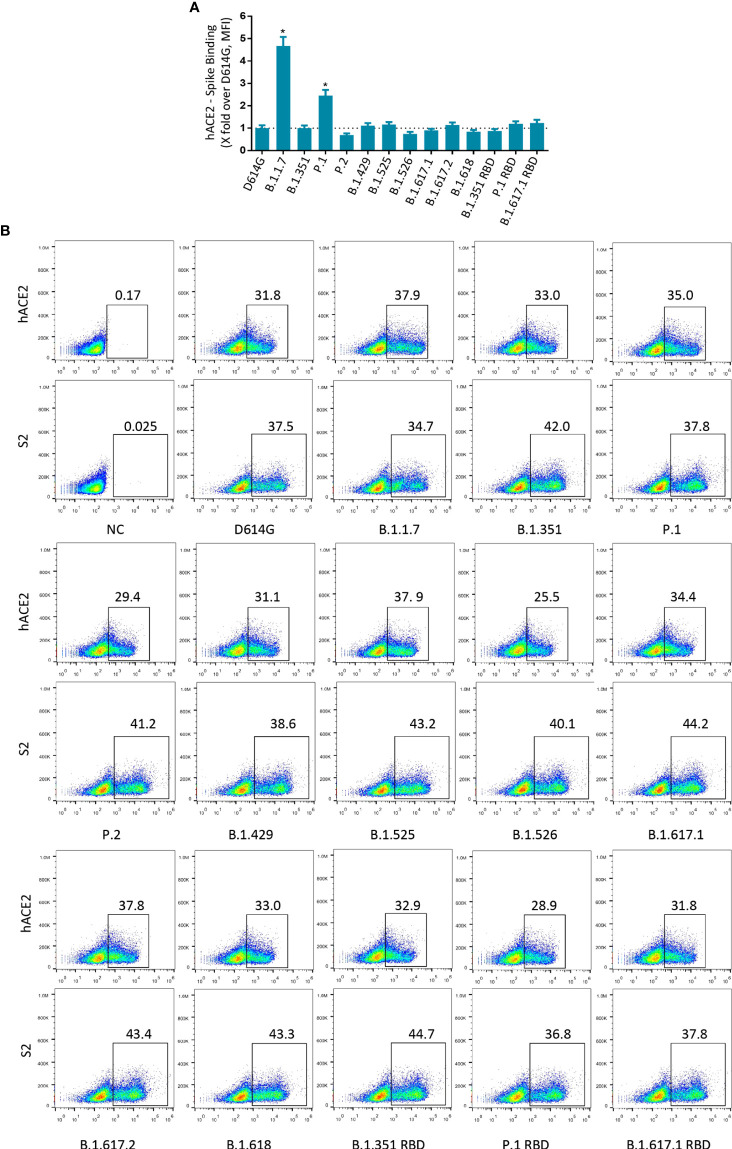
Binding of SARS-CoV-2 variants S protein to recombinant hACE2. **(A)** The fold changes in binding activity of SARS-CoV-2 variants S protein to recombinant hACE2, measured by mean fluorescence intensity (MFI). Recombinant hACE2 protein binding percentages were calculated by the ratio between variants over D614G MFI normalized relative to that of S2 specific antibody. All MFI values were weighted by multiplying the number of positive cells in the selected gates. **(B)** 293T cells transiently expressing SARS-CoV-2 variants S proteins were incubated with recombinant hACE2 protein or anti-SARS-CoV-2 spike (S2 subunit) antibodies for 1 h. After being washed, cells were incubated with Alexa Flour 488-conjugated anti-human IgG Fc or fluorescein isothiocyanate (FITC)-conjugated anti-mouse IgG secondary antibodies. A p-value less than 0.05 was defined as statistically significant (p < 0.05 [*]). Experiments were done at least twice.

SARS-CoV-2 RBD mediates the binding of the virus to the ACE2 receptor, which is the main determinant of the host range. Most mutations are deleterious for ACE2 binding, but some mutations are well tolerated and even enhance ACE2 binding (29). Next, we tested the binding avidity of variant S protein to ACE2 receptor. 293T cells expressing variant S protein were incubated with recombinant hACE2 and detected by flow cytometry. The mean fluorescence intensity (MFI) values of B.1.1.7 and P.1 were higher than those of D614G (**Figures 3A, B** and **Figures S3A, B**). However, the MFI values of other variants of S protein did not change significantly.

### Immune Escape of SARS-CoV-2 Variants to Neutralizing Monoclonal Antibodies

**Figure 4:**
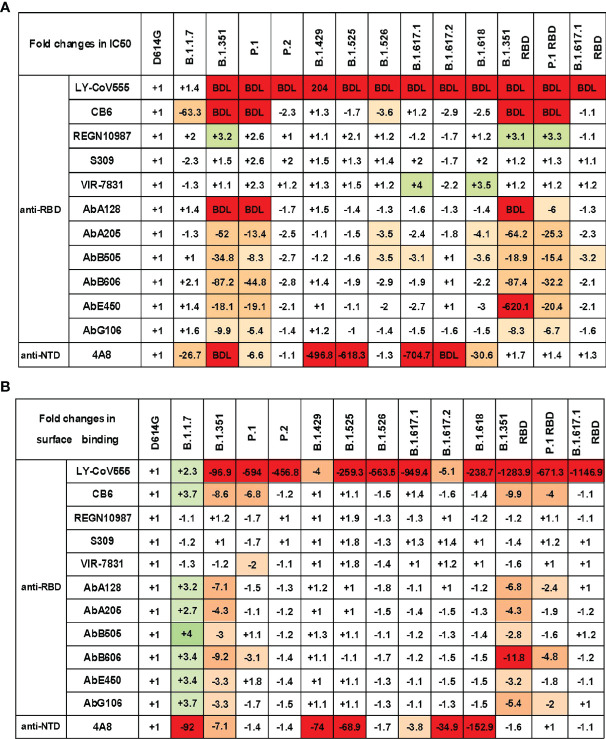
SARS-CoV-2 variant S pseudoviruses reduce neutralization and binding of mAbs. **(A)** The fold changes in neutralizing activity (IC50) of the mAbs against SARS-CoV-2 variants pseudovirus relative to D614G. **(B)** The fold changes in binding activity [mean fluorescence intensity (MFI)] of the mAbs against SARS-CoV-2 variants pseudovirus relative to D614G. “−” represents the decrease of sensitivity to the antibody, and “+” represents the increase of sensitivity to the antibody. The values marked in red indicate that sensitivity decreased at least 3-fold for IC50 or 2-fold for MFI, while those in green indicate that sensitivity increased at least 3-fold for IC50 or 2-fold for MFI. BDL indicates neutralizing activity below the detection limit. Experiments were done once.

To study the impact of SARS-CoV-2 variants on the antigenicity, we first measured the neutralization activity of 12 previously characterized neutralizing mAbs on pseudovirus infection in Vero cells (**Figure S4**). The neutralization profiles are shown in **Figure 4A**. Compared with the SARS-CoV-2 D614G reference strain, most of those anti-RBD mAbs showed varyingly decreased neutralizing activities to pseudovirus carrying the variant S protein. Specifically, B.1.1.7 decreased the sensitivity to mAb CB6, while P.2, B.1.429, B.1.525, and B.1.617.2 predominately decreased the sensitivity to mAb LY-CoV555. In addition, B.1.526, B.1.617.1, and B.1.618 became highly resistant to many mAbs, including LY-CoV555, CB6, AbA205, and AbB505. It is worth noting that B.1.351 and P.1 had the greatest immune escape ability on the tested neutralizing mAbs. Both of these variants reduced the sensitivity to mAbs LY-CoV555, CB6, AbA128, AbA205, AbB505, AbB606, AbE450, and AbG106. The neutralization activities of LY-CoV555, CB6, and AbA128 to B.1.351 and P.1 were below the detection limit (BDL). Previous studies have reported that SARS-CoV-2 RBD mutation could cause the decrease of neutralization activity of some mAbs (30). To verify our assay system, we also tested the same panel of mAbs against the pseudoviruses carrying just the mutations in the RBD region of B.1.351, P.1, and B.1.617.1. The results of B.1.351, P.1, and B.1.617.1 pseudoviruses were generally consistent with those of pseudoviruses with only RBD mutation. These findings indicate that the immune escape of B.1.351, P.1, and B.1.617.1 might mainly be mediated by mutations in the RBD region of SARS-CoV-2. It is worth noting that the mAbs REGN10987, S309, and VIR-7831 have a strong neutralization effect on almost all variants.

We also assessed the neutralization activity of the anti-NTD mAb 4A8 against SARS-CoV-2 variants (**Figure 4A**). B.1.1.7, B.1.351, P.1, B.1.429, B.1.525, B.1.617.1, B.1.617.2, and B.1.618 pseudoviruses showed various reductions in the sensitivity to 4A8. However, B.1.351 RBD, P.1 RBD, and B.1.617.1 RBD mutation pseudoviruses showed minimal change in neutralization activity. Therefore, the antibody targeting SARS-CoV-2 NTD can also be easily breached by certain mutations in the NTD domain of variant S protein, and the immune escape site has little to do with the RBD region.

The impaired binding affinity between the mAb and S protein is a major mechanism for SARS-CoV-2 immune escape (25). To study the correlation between variant immune escape and antibody affinity, we detected the binding affinity of mAbs to S protein expressed on 293T cells (**Figure 4B** and **Figure S5**). We found that the neutralization ability of antibodies has a strong correlation with binding affinity. In general, the weakening of the neutralization ability of mAbs is related to its decreased affinity with S protein.

### Altered Reactivity of SARS-CoV-2 Variants to Inactivated Virus Vaccine Sera

**Figure 5:**
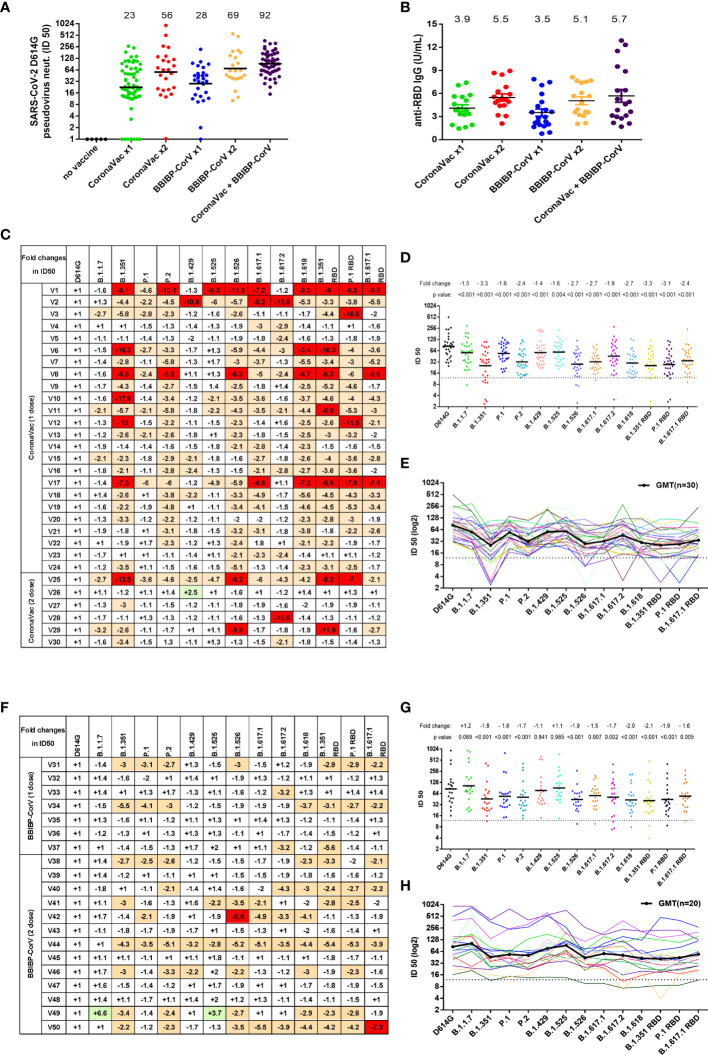
Neutralization activity to SARS-CoV-2 variants conferred by vaccine sera (CoronaVac or BBIBP-CorV). **(A)** Neutralization ID50 values of the vaccinated sera against SARS-CoV-2 D614G reference pseudovirus. The black horizontal lines and the numbers over the lines indicate geometric mean titers. **(B)** Anti-SARS-CoV-2 RBD IgG antibody levels were measured by a quantitative ELISA against the wild-type RBD antigen. ELISAs were performed in duplicate, and average values were used. The black horizontal lines and the numbers over the lines indicate the average values of anti-SARS-CoV-2 RBD IgG. **(C, F)** SARS-CoV-2 variants reduced neutralization sensitivity to CoronaVac vaccine sera **(C)** or BBIBP-CorV vaccine sera **(F)**. Fold changes of vaccine sera neutralization activity (ID50) between variants and D614G reference strain pseudoviruses, depicted in a heat map. “−” represents the decrease of sensitivity of vaccine sera, and “+” represents the increase of sensitivity of vaccine sera. The values are marked in red, indicating that sensitivity is decreased at least 2-fold, while those in green indicate that sensitivity is increased at least 2-fold. **(D, E, G, H)** Neutralization ID50 values of the vaccinated sera [CoronaVac **(D, E)** or BBIBP-CorV **(G, H)**] against SARS-CoV-2 variant pseudoviruses. The geometric mean titer against each variant is indicated by a black horizontal line in panels **(D, G)** and a black curve in panels **(E, H)** The fold changes of ID50 between variant and D614G pseudoviruses are illustrated by the overall average at the top in **(D, G)**. The dashed line represents the initial dilution of vaccine sera. Neutralization activity is defined as the percent reduction in luciferase activity relative to the virus control wells (virus + cells).

We next investigated the resistance of variant pseudovirus on neutralization activity of vaccine sera obtained from 179 participants after receiving one or two doses of inactivated virus vaccine—CoronaVac or BBIBP-CorV (**Figure S6**). We first measured the neutralization titers of serum samples against the reference D614G pseudovirus and found that the majority of the vaccinated participants produced neutralizing antibodies (**Figure 5A**). The geometric mean neutralizing titers of one dose of CoronaVac, two doses of CoronaVac, one dose of BBIBP-CorV, two doses of BBIBP-CorV, and CoronaVac+BBIBP-CorV were 23, 56, 28, 69, and 92, respectively. On the other hand, undetectable neutralization activities were seen in 7 out of 65 serum samples from subjects receiving only one dose of CoronaVac, in 1 out of 24 serum samples from subjects receiving two doses of CoronaVac, and in 1 out of 29 serum samples from subjects receiving one dose of BBIBP-CorV. Consistent with the neutralization effect of serum samples on pseudoviruses, the average anti-RBD IgG levels of one dose of CoronaVac, two doses of CoronaVac, one dose of BBIBP-CorV, two doses of BBIBP-CorV, and CoronaVac+BBIBP-CorV were 3.9, 5.5, 3.5, 5.1, and 5.7 U/ml, respectively (**Figure 5B**).

Next, we selected 30 CoronaVac serum samples and 20 BBIBP-CorV serum samples to test the immune escape of SARS-CoV-2 variants. For CoronaVac serum samples, when compared with SARS-CoV-2 D614G, the neutralization activity to most variants was somewhat decreased (**Figures 5C–E**). The average reduction of neutralization activity in serum samples of CoronaVac was 1.5-fold against B.1.1.7, 3.3-fold against B.1.351, 1.6-fold against P.1, 2.4-fold against P.2, 1.4-fold against B.1.429, 1.6-fold against B.1.525, 2.7-fold against B.1.526, 2.7-fold against B.1.617.1, 1.9-fold against B.1.617.2, and 2.7-fold against B.1.618. Consistent with the findings for anti-SARS-CoV-2 RBD mAbs, B.1.351 RBD, P.1 RBD, and B.1.617.1 RBD pseudoviruses showed similar immune escape with B.1.351, P.1, and B.1.617.1, respectively. Although the neutralization activities of vaccine sera against the variants were decreased, most of the variants still could be effectively neutralized, including the strong escaping strains B.1.351, P.1, P.2, B.1.526, B.1.617.1, and B.1.617.2. We also observed that there is significant variation in resistance to immunity escape among the vaccinated sera of participants. Some individuals showed greater reductions in neutralizing certain variants, most noticeably B.1.351, B.1.526, B1.617.1, and B.1.618, as well as B.1.351 RBD and P.1 RBD (**Figure 5C** and **Figure S6**).

For BBIBP-CorV vaccine serum samples, when compared with the neutralization activity to D614G, the sera had equal activity in neutralizing B.1.1.7, B.1.429, and B.1.525 (**Figures 5F–H**), while the neutralization efficiencies were remarkably decreased to B.1.351 (1.8-fold), P.1 (1.6-fold), P.2 (1.7-fold), B.1.526 (1.9-fold), B1.617.1 (1.5-fold), B.1.617.2 (1.7-fold), and B.1.618 (2.0-fold). Likewise, B.1.351 RBD, P.1 RBD, and B.1.617.1 RBD pseudoviruses showed a similar immune escape as B.1.351, P.1, and B.1.617.1, respectively.

### Effects of Cathepsin Inhibitor and Endocytosis Inhibitors on SARS-CoV-2 Entry

**Figure 6:**
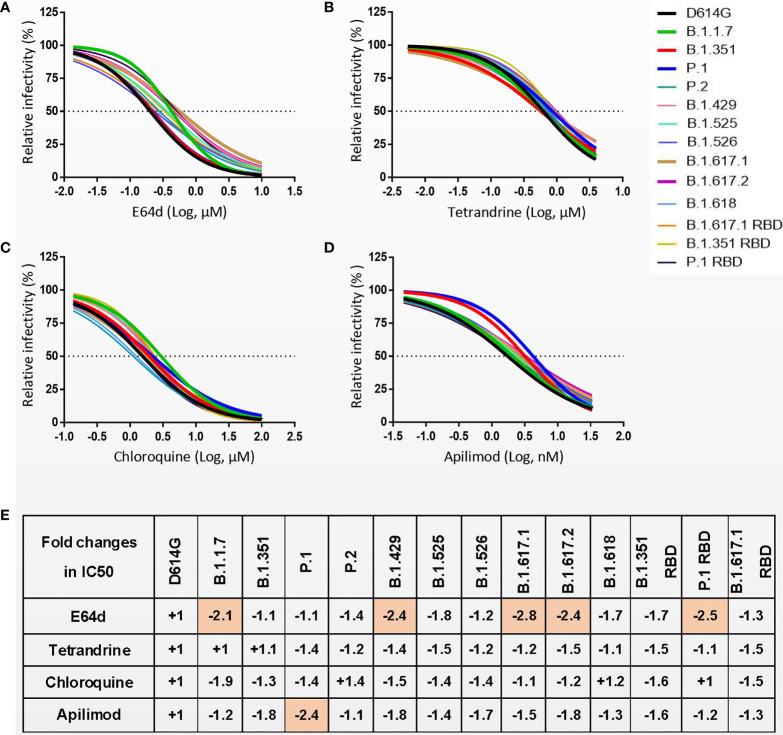
**(A–D)** 293T-hACE2 cells were pretreated with different concentrations of cathepsin inhibitor E64d **(A)** or endocytosis inhibitors Tetrandrine **(B)**, Chloroquine **(C)**, and Apilimod **(D)**, and then infected with SARS-CoV-2 S pseudoviruses. The luciferase activity was measured 24 h postinoculation. **(E)** The fold changes in half maximal inhibitory concentration (IC50) of inhibitors against SARS-CoV-2 variants pseudovirus relative to D614G. “-” represents the decrease of sensitivity to the inhibitor, and “+” represents the increase of sensitivity to the inhibitor. The values marked in red indicate that sensitivity decreased at least 2-fold. Experiments were done in 4 replicates and repeated at least twice. One representative is shown with error bars indicating SEM.

SARS-CoV-2 S protein priming in cells can be activated by endosomal cysteine proteases cathepsin B and L (CatB/L) (6, 8). Similarly, SARS-CoV-2 enters target cells mainly through endocytosis, and inhibiting the maturation and transport of endosomes can inhibit viral infections (6). To investigate whether the entry of SARS-CoV-2 S variants can be inhibited by cathepsin inhibitor or endocytosis inhibitor, 293T-hACE2 cells were treated with either cathepsin inhibitor E64d or endocytosis inhibitors Chloroquine, Tetrandrine, and Apilimod and then evaluated their effect on virus entry (**Figures 6A–E** and **Figures S7A–E**). All inhibitors were found to reduce the entry of variants of pseudovirus on 293T-hACE2 cells in a dose-dependent manner. The entry of B.1.1.7, B.1.429, B.1.617.1 B.1.617.2, and P.1 RBD pseudoviruses were slightly less sensitive to blockade by E64d as compared to D614G (**Figure 6E**). P.1 pseudovirus was slightly less sensitive to blockade by Apilimod as compared to D614G (**Figure 6E**). However, endocytosis inhibitors Chloroquine and Tetrandrine had similar blocking effects on all SARS-CoV-2 variants (**Figure 6E**). These results suggest that cathepsin inhibitors and endocytosis inhibitors will be active against SARS-CoV-2 variants.

## Discussion

As the COVID-19 pandemic persists, new SARS-CoV-2 variants will continue to emerge. Some mutations lead to changes in viral infectivity or may be able to escape neutralizing antibodies. In this study, we constructed the major circulating SARS-CoV-2 variants and identified the effects of these variants on virus infectivity, antigenicity, and sensitivity to inhibitors. The present study shows that, compared with the D614G strain, the infectivity of most variants was changed in mammalian cells. In addition, the antigenicity of some variants has also changed, resulting in a significant reduction in the neutralizing activity of a variety of RBD or NTD-targeting mAbs. Serum samples from individuals vaccinated with CoronaVac or BBIBP-CorV also showed the varyingly decreased neutralizing activity to many SARS-CoV-2 variants. Close monitoring of the major circulating SARS-CoV-2 variants can better control the spread of the virus, and it is also important for the development of neutralizing antibodies and vaccines.

The rapid spread of SARS-CoV-2 variants in the population raises the possibility that these viruses might exhibit altered infectivity or infection dynamics. After the SARS-CoV-2 outbreak, the D614G strain quickly replaced the original strain and became the dominant variant. Studies show that the D614G substitution leads to the enhancement of viral replication and infectivity in host cells (19, 20). Subsequently, several new SARS-CoV-2 variants were reported to spread rapidly in various countries and regions. In particular, B.1.17, B.1.351, P.1, and B.1.617.2 all showed higher infectivity than the original strain in the real world (31–34). In our study, the infectivity of the major circulating SARS-CoV-2 variants had no significant difference in 293T-hACE2, 293T-hACE2-TMPRSS2, Caco2-hACE2, and Vero cells. But the infectivity of some variants was significantly enhanced in Caco2, Huh7, A549, and H1299 cells. Recently, Zhang et al. (28) showed that the fusion activity among D614G, B.1.1.7, B.1.351, P.1, B.1.617.1, and B.1.617.2 had no significant differences when spike and ACE2 were transfected at high levels. However, B.1.617.2 can enter host cells expressing low levels of ACE2 more effectively than other variants. We also found that the infectivity of B.1.617.2 was significantly enhanced in ACE2 low-expression Caco2, Huh7, A549, and H1299 cells. This suggests that B.1.617.2 is more likely to infect less susceptible cells and then replace the previously dominant variants, leading to a global pandemic.

Priming of SARS-CoV-2 S proteins by host cell proteases is crucial for virus entry into cells (8). Insertion of a furin cleavage site with four amino acid motifs (PRRA) at the S1/S2 junction of SARS-COV-2 S protein can affect the cleavage of the viral S protein (35). The P681H, P681R, and Q677H mutation sites in the variants are located near the furin cleavage site, which may affect the cleavage of S protein (35–37). In fact, B.1.617.2 contains the P681R mutation, which results in enhanced cleavage of the S protein in cells, which may account for the enhanced infectivity of B.1.617.2 in some susceptible cells.

The main receptor for SARS-CoV-2 infection of host cells is ACE2, but several other receptors have been identified that promote SARS-CoV-2 infection (38–41). Some of these receptors affect SARS-CoV-2 infection by binding to sites outside the RBD region. Wang et al. (41) found that H1299 cells do not express the ACE2 receptor but highly express tyrosine-protein kinase receptor UFO (AXL), and it can bind to the SARS-CoV-2 NTD to mediate viral infection. In our study, most of the variants have NTD mutations, which may lead to changes in the binding ability of SARS-CoV-2 NTD to the AXL receptor, thereby affecting the ability of the virus to infect H1299 cells.

Hoffmann et al. (16) reported that pseudoviruses bearing the S protein of variants exhibit similar entry kinetics in Vero cells. In this study, we found that SARS-CoV-2 variants exhibited similar infectivity in Vero cells at an early stage. However, in Caco2-hACE2, 293T-hACE2, and 293T-hACE2-TMPRSS2 cells, B.1.1.7, B.1.351, and B.1.617.2 exhibit higher infection efficiency than D614G in shorter incubation time. These data suggest that some variants infect target cells much faster than the original strain, which may be the reason for their enhanced transmission ability.

At present, neutralizing mAbs and vaccines against SARS-CoV-2 are mainly developed against SARS-CoV-2 strains in the early stage of the pandemic (42–48). Our findings suggest that most of the circulating variants possess certain immune escape abilities to mAbs. Anti-RBD mAbs are divided into four categories according to the antibody recognition pattern and epitope structural characteristics (49). In this study, the antibody CB6 belongs to class I anti-RBD antibodies, which blocks ACE2 and bind only to “upper” RBDs. Previous studies have shown that the SARS-CoV-2 K417N/T mutation can lead to reduced neutralizing activity of CB6 (25). In our study, B.1.351, P.1, B.1.351 RBD, and P.1 RBD carrying the K417 mutation exhibited significant immune escape from CB6. LY-CoV555 is a class II anti-RBD antibody that blocks ACE2 and binds to both “upper” and “down” RBDs. LY-CoV555 has immune escape against almost all variants except D614G and B.1.17. The immune escape of some variants against LY-CoV555 may be related to the E484K/A mutation (24, 50). The mAbs REGN10987, S309, and VIR-7831 have strong neutralizing effects on almost all variants. These three antibodies belong to class III anti-RBD antibodies that bind outside the ACE2 site and recognize “up” and “down” RBDs (49), indicating that the neutralizing effect of antibodies is less affected when targeting these sites. Furthermore, 4A8 targets the SARS-CoV-2 NTD domain, and its neutralizing activity can be affected by SARS-CoV-2 Y144del, LAL242-244del, and R246I mutations (24, 25). Our studies show that its neutralizing activity is disrupted by certain mutations in the NTD domain, whereas the immune escape site is less related to the RBD region.

Similar to the neutralizing activity of antibodies, LY-CoV555 had reduced binding capacity for almost all variants except D614G and B.1.17. The binding affinity of mAbs REGN10987, S309, and VIR-7831 to variant S protein did not change significantly. The remaining anti-RBD mAbs had reduced binding ability to B.1351 or P.1. Most antibodies with reduced neutralizing activity to variant pseudoviruses also have reduced binding affinity, indicating that the antibody binding affinity is closely related to its neutralizing activity. The binding ability of these anti-RBD antibodies to the S protein is closely related to mutations in the RBD region. However, the binding ability of the 4A8 antibody to the S protein was associated with NTD mutations.

For neutralizing activity of CoronaVac and BBIBP-CorV vaccine sera, we found that most vaccine recipients were able to produce neutralizing antibodies and had the ability to neutralize the virus. Previous studies have shown that sequential booster immunizations with mRNA vaccines or subunit vaccines after two doses of inactivated vaccine are more effective than homologous vaccines (51, 52). Indeed, mixed vaccination of CoronaVac and BBIBP-CorV vaccine resulted in higher levels of anti-RBD IgG antibodies and stronger virus neutralization than a single vaccination regimen. For neutralization activity of CoronaVac and BBIBP-CorV vaccine sera against different variants, our study is consistent with the results of recent reports using mRNA vaccine or inactivated vaccine sera (17, 18, 24, 53–55). We found that some variants, such as B.1.1.7, B.1.429, and B.1.525, have weaker immune escapes against CoronaVac or BBIBP-CorV vaccine sera. However, some variants, such as B.1.351, P.1, P.2, B.1.526, B.1.617.1, B.1.617.2, and B.1.618, have strong immune escapes against vaccine sera. Although the neutralizing activity to some SARS-CoV-2 variants was decreased, CoronaVac and BBIBP-CorV serum samples still reserved effective neutralizing abilities to all variants.

Proteolytic activation of S protein is an essential step for SARS-CoV-2 to enter target cells (6, 8, 56, 57). In this study, we confirmed that SARS-CoV-2 spread depends on cathepsin activity and further showed that the S protein-driven entry of the SARS-CoV-2 variant is effectively blocked by endosomal cysteine proteases CatB/L inhibitor. Ou et al. (6) showed that SARS-CoV-2 can enter host cells through endocytosis. The early-to-late endosomal maturation is regulated by PI(3,5)P_2_ (58, 59). Inhibition of PI(3,5)P_2_ synthase PIKfyve or the downstream effector TPC_2_ can significantly reduce SARS-CoV-2 entry (6). Consistent with these findings, entry of all SARS-CoV-2 variants was effectively blocked by targeted endocytosis inhibitors Chloroquine, Tetrandrine, and Apilimod. Therefore, drugs targeting SARS-CoV-2 protease activity or endocytosis pathway are a commonly effective way in treating SARS-CoV-2 regardless of different mutations in the spike protein causing immune evasion.

Recently, a new SARS-CoV-2 variant, Omicron (B.1.1.529), emerged in South Africa, rapidly replacing B.1.617.2 and becoming the dominant strain. A striking feature of this variant is the large number of mutations in the S protein, most of which are located in the RBD region (60), which poses a threat to the efficacy of current COVID-19 vaccines and antibody therapies. Current studies have shown that B.1.1.529 is not only resistant to neutralization by sera from convalescent patients with COVID-19 but also significantly resistant to sera from vaccinated individuals (50, 61–63). Our published data (64) also demonstrate that pseudoviruses carrying the Omicron S protein have strong immune evasion capabilities against mAbs and vaccine sera. However, Omicron is still dependent on hACE2 for entry into target cells, and its S protein maintains a strong interaction with hACE2.

In summary, we demonstrate that the infection efficiency of pseudovirus with SARS-CoV-2 variant S protein has changed in some target cells; especially, the B.1.617.2 strain is more infectious in less susceptible cells to the original strain, and the virus infection process can be completed in a shorter time. In addition, our data suggest that SARS-CoV-2 variants may compromise the therapeutic effect of neutralizing antibodies or reduce the protective effect of vaccines. Our findings highlight the need to strengthen virus surveillance and assess the effectiveness of current antibodies and authorized vaccines against emerging SARS-CoV-2 variants. Meanwhile, increasing the proportion of people vaccinated with effective SARS-CoV-2 vaccines is the key strategy for reducing the emergence of new variants and ending the pandemic of COVID-19.

## Methods

### Serum Samples

Vaccine sera were obtained from the participants who received CoronaVac and/or BBIBP-CorV vaccine at Suzhou Science and Technology Town Hospital in Suzhou City, Jiangsu Province, China, and stored at −80°C until use. Detailed vaccination information is provided in **Table S1**. The study was approved by the Institutional Review Board of Suzhou Science and Technology Town Hospital (IRB2021006). All subjects provided informed consent for the testing of their serum samples in this study.

### Monoclonal Antibodies

All mAbs used in the present study were screened and provided by AtaGenix Company (Wuhan, China). Antibodies developed by other organizations, including LY-CoV555, CB6, REGN10987, S309, VIR-7831, and, 4A8, were produced based on the sequences published in Protein Data Bank (PDB). All antibodies were repackaged and stored at −80°C to avoid inconsistent results caused by repeated freeze–thaw cycles.

### Cell Lines

293T (human, kidney), Caco-2 (human, colon), Huh7 (human, liver), A549 (human, lung), H1299 (human, lung), and Vero (African green monkey, kidney) cells were obtained from American Type Culture Collection (ATCC, Manassas, VA, USA). 293T-hACE2, 293T-hACE2-TMPRSS2, and Caco-2-hACE2 were produced by lentiviral mediated gene transduction. All cells were cultured in Dulbecco’s modified Eagle medium (DMEM) (HyClone, Logan, UT, USA) containing 10% fetal bovine serum (FBS) (Gibco, Grand Island, NY, USA) and 100 U/ml of penicillin-streptomycin (Gibco).

### Construction of SARS-CoV-2 Variant S Protein Expression Plasmids

The SARS-CoV-2 Wuhan-1 S gene (GenBank: MT_613044) was obtained from GenScript Biotech Corporation (Piscataway, NJ, USA). To effectively incorporate S protein into pseudovirus, the last 19 amino acids in the cytoplasmic tail of S protein were removed according to previously reported methods (65). To create variant S protein expression plasmids, point mutation on defined sites of the S gene was carried out using the site-directed mutagenesis kit (KOD). Subsequently, multiple PCR fragment amplification utilizing oligonucleotides containing mutation and overlapping sequence was performed for each desired mutation. Finally, overlapping fragments were assembled to produce all the mutations of each strain. The primers used to construct mutants are listed in **Table S2**.

### Production of SARS-CoV-2 S Protein Pseudoviruses

Pseudoviruses with SARS-CoV-2 S protein were produced according to the methods reported in our previous study (66). Briefly, 293T cells transfected to express SARS-CoV-2 S protein under study were inoculated with G*ΔG-VSV dual reporter virus (kindly provided by UltraImmune Inc.). After 6 h of virus infection, the cells were gently washed twice with phosphate-buffered saline (PBS) to remove residual G*ΔG-VSV virus. Viral supernatant was collected at 24 or 48 h postinoculation and centrifuged at 4,000*g* for 5 min to remove cell debris.

The pseudovirus particles were quantified by RT-qPCR. Viral RNA Mini kit was used to extract virus RNA from 200 μl of pseudoviruses containing supernatant. Then, the viral RNA served as a template and reversed to cDNA. Virus particle quantification was performed by qPCR using FastStart Essential DNA Green Master (Roche, Basel, Switzerland). The copy numbers of virus particles were calculated according to VSV-P gene. Primers used to calculate pseudoviral particles are listed in **Table S3**.

### Pseudovirus Infection Assay

Pseudoviral particles were normalized to the same amount using quantitative RT-PCR. Before virus infection, 2 × 10^4^ target cells were seeded into each well of 96-well plates. Then, 100 μl of media containing pseudoviruses was inoculated into the cells. After incubation for 24 h, cells were lysed with passive lysis buffer (Promega, Madison, WI, USA) for 10 min, and then the luciferase activity was measured by the Luciferase Assay System (Promega). In order to analyze the entry of the pseudovirus into the target cells at indicated time points, the medium was removed at different time points, and the cells were washed with PBS to remove the remaining virus. Then 100 μl of fresh medium was added, and culture was continued for 24 h.

### Detection of SARS-CoV-2 Variant S Protein

To detect the expression of S protein in cells, 293T cells were transfected with expression plasmids encoding SARS-CoV-2 variant S protein. After 40 h of transfection, cells were lysed by radioimmunoprecipitation assay (RIPA) Lysis Buffer (Beyotime, Shanghai, China) for 30 min on ice. To detect the cleavage of S protein on pseudoviruses, 1 ml of the SARS-CoV-2 pseudoviruses was loaded by 6% PEG8000 and shaken on the ice for 8 h. To pellet down pseudoviruses, the mixture was centrifuged at 10,000*g* for 2 h at 4°C. Next, the concentrated virus particles were resuspended in 50 μl of RIPA Lysis Buffer. The protein samples were heated at 95°C for 10 min. Then protein samples were separated in a 10% sodium dodecyl sulfate–polyacrylamide gel electrophoresis (SDS-PAGE) gel (Beyotime) and transferred onto polyvinylidene difluoride (PVDF) membranes (Millipore, Billerica, MA, USA). After protein transfer, the PVDF membranes were blocked by 5% milk for 1 h and then incubated overnight with primary antibodies. The next day, the PVDF membranes were incubated with secondary antibodies and visualized by the ChemiDoc MP system (Bio-Rad, Hercules, CA, USA). The following antibodies were used: mouse anti-SARS-CoV-2 spike (S2 subunit) mAb [1A9] (Genetex, Irvine, CA, USA; 1:2,000), mouse anti-VSV matrix protein (Kerafast, Boston, MA< USA; 1:2,500), GAPDH mAb (ProteinTech, Chicago, IL, USA; 1:2,000), and horseradish peroxidase linked anti-mouse IgG antibody (CST, Danvers, MA, USA; 1:5,000).

### Enzyme-Linked Immunosorbent Assay

Anti-SARS-CoV-2 S-RBD IgG was detected using Human SARS-CoV-2 S-RBD IgG ELISA Kit (AtaGenix) according to the manufacturer’s instructions. Serum samples were diluted at 1:2,000 with dilution buffer. The level of anti-RBD antibodies in the serum sample is represented by the concentration of the positive control antibody. One unit per ml (U/ml) represents the equivalent neutralization capacity of 1 ng/ml of control antibody.

### Flow Cytometric Assessment of S Proteins Expression and the Binding of Monoclonal Antibodies to Cell Surface-Expressed SARS-CoV-2 S Proteins

293T cells were transfected with expression plasmids encoding SARS-CoV-2 S proteins and cultured for 36 h. Cells were digested with trypsin and washed twice with 1 ml of staining buffer (PBS containing 2% FBS). First, cells were incubated with mouse anti-SARS-CoV-2 spike (S2 subunit) antibody (Genetex, 1 μg/ml), neutralizing mAbs (AtaGenix, 0.2 μg/ml), or recombinant hACE2 protein (Sino Biological, Beijing, China; 1 μg/ml) at 4°C for 1 h. After being washed, cells were incubated with Alexa Flour 488-labeled anti-human IgG Fc (BioLegend, San Diego, CA, USA) and/or PE-labeled anti-mouse IgG (BioLegend) secondary antibodies for 1 h. After being washed, the cells were resuspended and analyzed using the Attune™ NxT Flow Cytometer (Thermo Fisher Scientific, Waltham, MA, USA).

### Pseudovirus Neutralization Assays

The effects of the neutralizing antibodies and vaccinated sera on the entry inhibition of pseudoviruses were examined by detecting the reduction in luciferase gene expression (24, 30, 67). In brief, Vero or 293T-hACE2 cells were seeded in a 96-well plate at a concentration of 2 × 10^4^ cells per well. To test the neutralization activity of the neutralizing antibodies, serial 5-fold dilution of samples were prepared at an initial concentration of 1 μg/ml and then incubated with 1,000 TCID50 pseudoviruses at 37°C for 1 h, and the mixture was added to Vero cells. To test the neutralization activity of vaccine sera, serial 3-fold dilution of samples were prepared with the starting dilution of 1:12 (all samples except V43–V50) or 1:20 (samples V43–V50) and then incubated with 1,000 TCID50 pseudoviruses at 37°C for 1 h, and the mixture was added to 293T-hACE2 cells. After incubation for 24 h, the neutralization activity was quantified by measuring the luciferase activity in cell lysates. Neutralization activity was defined as the percentage of decrease in luciferase activity compared to the virus control wells (virus + cells).

### Effects of Protease and Endocytosis Inhibitors on Pseudovirus Entry

For experiments involving protease inhibitor (E64d, MCE, South Brunswick, NJ, USA) or endocytosis inhibitors (Chloroquine, MCE; Tetrandrine, Sigma, St. Louis, MO, USA; and Apilimod, MCE), 293T-hACE2 cells were pretreated with corresponding inhibitors for 2 h before pseudovirus infection. Then, SARS-CoV-2 variants of pseudoviruses were added to the cell culture wells. The infection efficiency of viruses was quantified 24 h post-infection by measuring the luciferase activity in cell lysates.

### Statistical Analysis

Half-maximal inhibitory concentration (IC50) or half-maximal inhibitory dilution (ID50) was defined as the RLU values that were reduced by 50% compared to the virus control wells (virus + cells). IC50 and ID50 were calculated by the equation of four-parameter dose inhibition response in GraphPad Prism 7. Vaccine sera, with neutralizing activity BDL, were assigned a value of 1 for geometric mean calculations and were considered as seronegative. The serum from healthy donors without vaccination was used as negative control and showed no detectable neutralization activity. The significance of neutralizing activities of serum samples against each variant pseudovirus relative to D614G was estimated using the Wilcoxon matched-pairs signed-rank test. Two-tailed p-values were reported, and p < 0.05 was defined as statistically significant.

## Data Availability Statement

The original contributions presented in the study are included in the article/**Supplementary Material**. Further inquiries can be directed to the corresponding authors.

## Ethics Statement

The studies involving human participants were reviewed and approved by the Institutional Review Board of Suzhou Science and Technology Town Hospital (IRB2021006). The patients/participants provided their written informed consent to participate in this study. Written informed consent was obtained from the individual(s) for the publication of any potentially identifiable images or data included in this article.

## Author Contributions

HT performed the experiments and wrote the manuscript. HT, XD, and FQ analyzed data. LG, ZW, FM, XZ, YS, and GH contributed to revise the manuscript and approved the final manuscript. XD and FQ were responsible for research design, strategy, and supervision. All authors listed have made a substantial, direct, and intellectual contribution to the work and approved it for publication.

## Funding

This work was supported by the National Natural Science Foundation of China (Grants 31800726 and 81773058), The Chinese Academy of Medical Sciences Initiative for Innovative Medicine (Grant CAMS-I2M, 2016-I2M-1-005), Key Research and Development Project of Zhejiang Province (No. 2021C03198), and National Grand Foreign Experts projects (G20190001633 and G20190001639).

## Conflict of Interest

The authors declare that the research was conducted in the absence of any commercial or financial relationships that could be construed as a potential conflict of interest.

## Publisher’s Note

All claims expressed in this article are solely those of the authors and do not necessarily represent those of their affiliated organizations, or those of the publisher, the editors and the reviewers. Any product that may be evaluated in this article, or claim that may be made by its manufacturer, is not guaranteed or endorsed by the publisher.

## Supplementary Material

Supplementary Figure 1 Structural diagram of mutation sites of variants S protein. The PDB number for the reference S protein structure is 7DDN. Each variant is shown from three perspectives, yellow represents the RBD region, green represents the NTD region, and turquoise represents the S2 region. Some mutation sites were not marked due to the lack of clear structural elucidation of these sites.

Supplementary Figure 2 Infectivity analysis of SARS-CoV-2 variants in target cells in the early stage, repeated experiments of **Figures 2B**.

Supplementary Figure 3 Binding of SARS-CoV-2 variants S protein to recombinant hACE2, repeated experiments of **Figures 3**.

Supplementary Figure 4 Neutralization of SARS-CoV-2 variants by mAbs, related to **Figures 4**. Pseudoviruses carrying the variant S proteins were tested against a series of dilutions of each mAb. Neutralization activity was defined as the percentage of decrease in luciferase activity compared to the virus control wells (virus + cells).

Supplementary Figure 5 Binding to cell surface expressed SARS-CoV-2 variants S proteins by neutralizing mAbs, related to **Figures 4**. SARS-CoV-2 variants S proteins were expressed on the surface of 293T cells, incubated with anti-SARS-CoV-2 spike antibody and neutralizing mAbs, followed by staining with Alexa Flour 488-conjugated anti-human IgG Fc and PE-conjugated anti-mouse IgG antibodies, and analyzed by flow cytometry. Experiments were done once. NC is 293T cells with mock transfection.

Supplementary Figure 6 Neutralization of SARS-CoV-2 variants by vaccine sera, related to **Figures 5**. Pseudoviruses carrying the indicated variant S proteins were tested against serial dilutions of vaccine sera [CoronaVac vaccine (V1-V30) or BBIBP-CorV vaccine (V31-V50)]. Neutralization activity was defined as the percent reduction in luciferase activity relative to the virus control wells (virus + cells).

Supplementary Figure 7 SARS-CoV-2 S pseudovirus entry into 293T-hACE cells can be blocked with endocytosis and protease inhibitors, repeated experiments of **Figures 6**.
