# Supplementary figures and images for: Multiple SARS-CoV-2 Variants Exhibit Variable Target Cell Infectivity and Ability to Evade Antibody Neutralization
Source: Front Immunol. 2022 Mar 16;13:836232. doi: 10.3389/fimmu.2022.836232 (PMC8966392; doi:10.3389/fimmu.2022.836232)

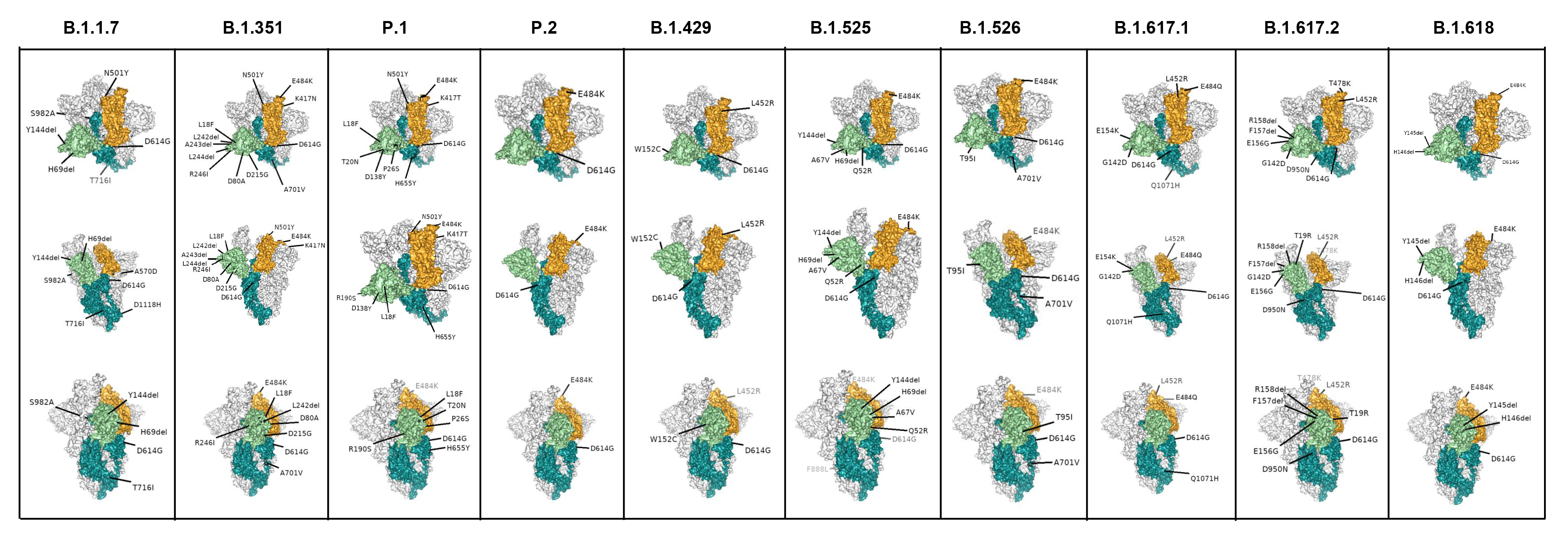

Supplement: Supplementary Figure 1 — Structural diagram of mutation sites of variants S protein. The PDB number for the reference S protein structure is 7DDN. Each variant is shown from three perspectives, yellow represents the RBD region, green represents the NTD region, and turquoise represents the S2 region. Some mutation sites were not marked due to the lack of clear structural elucidation of these sites. [file Image_1.tif]

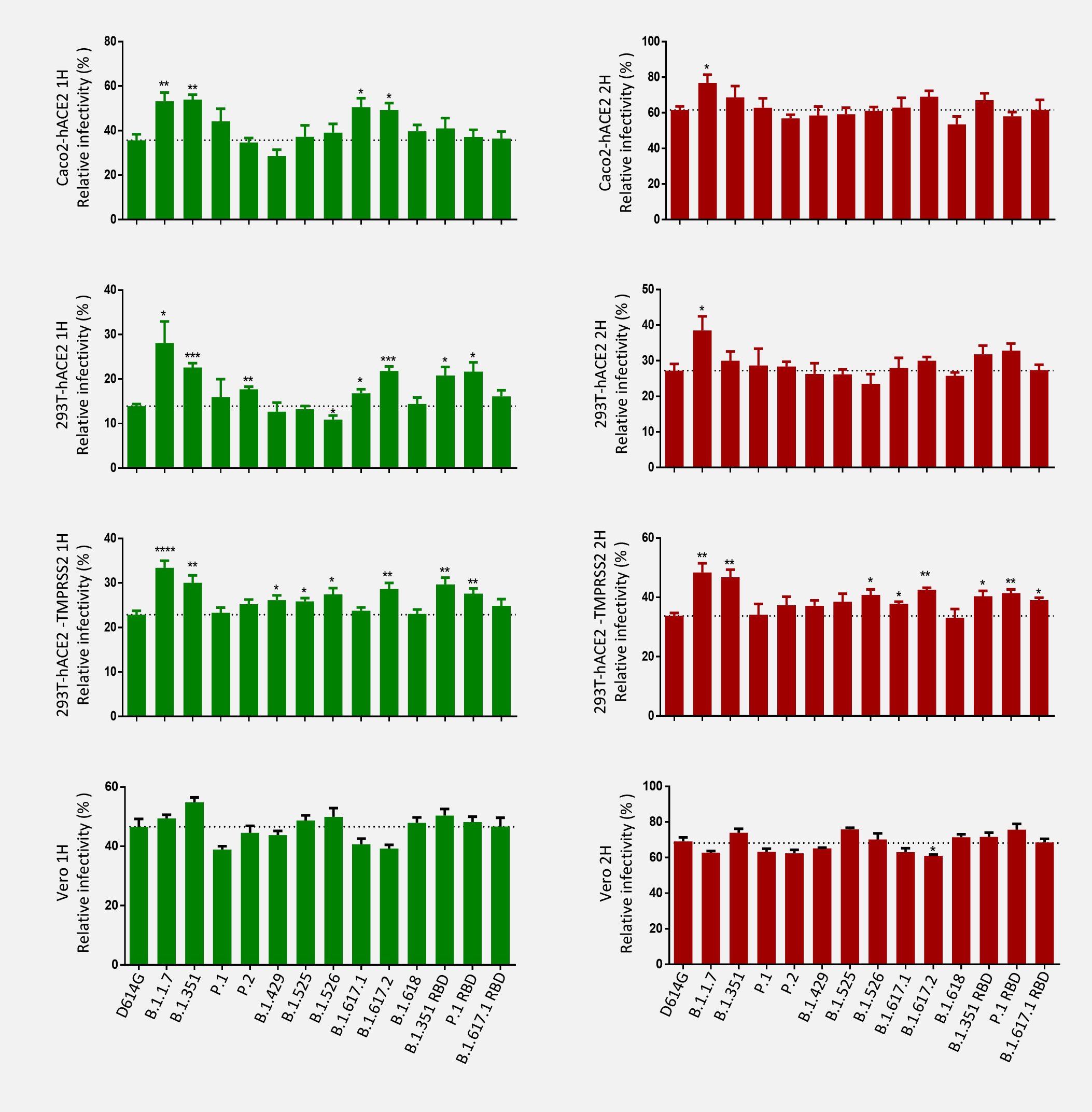

Supplement: Supplementary Figure 2 — Infectivity analysis of SARS-CoV-2 variants in target cells in the early stage, repeated experiments of Figures 2B . [file Image_2.tif]

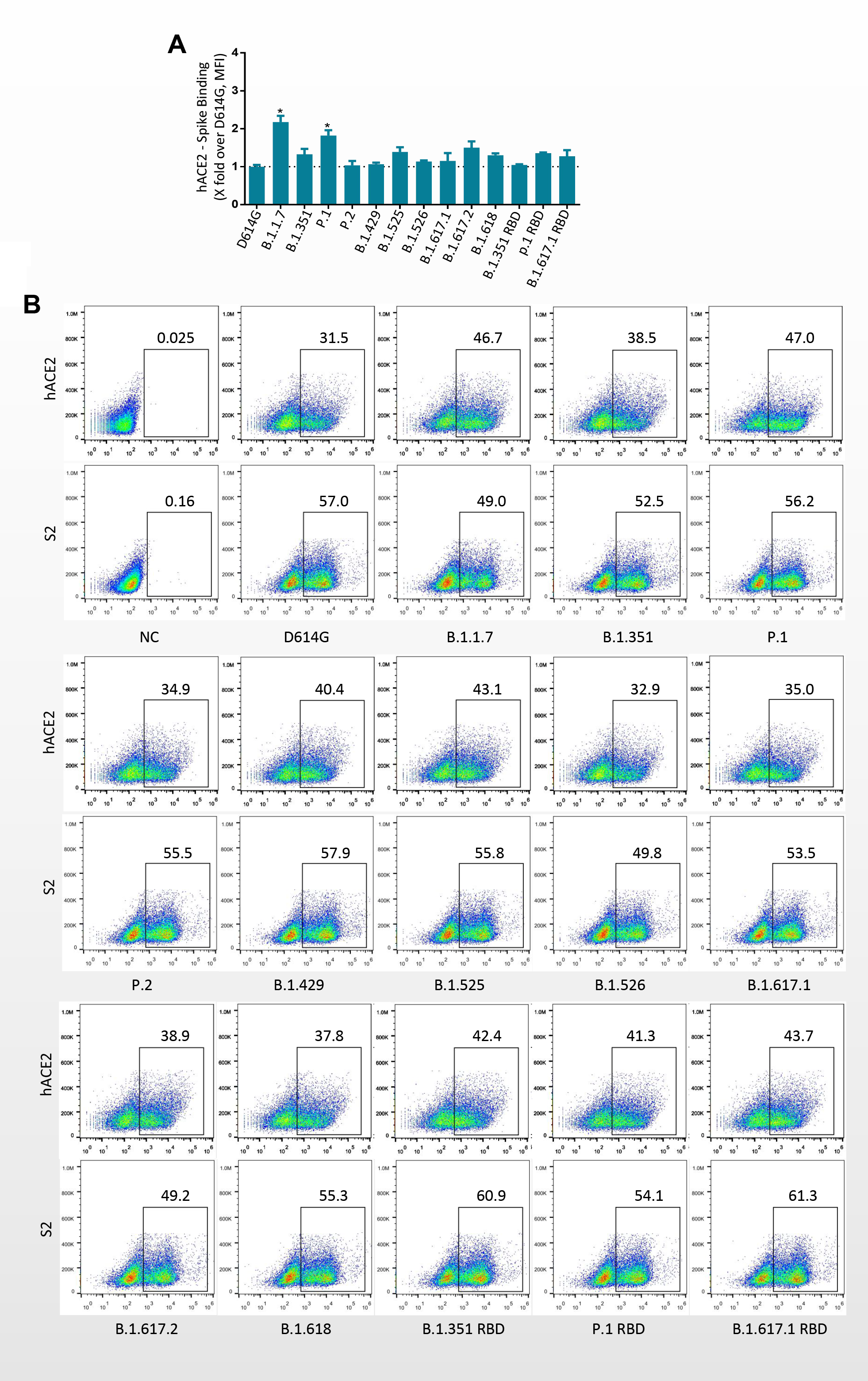

Supplement: Supplementary Figure 3 — Binding of SARS-CoV-2 variants S protein to recombinant hACE2, repeated experiments of Figures 3 . [file Image_3.tif]

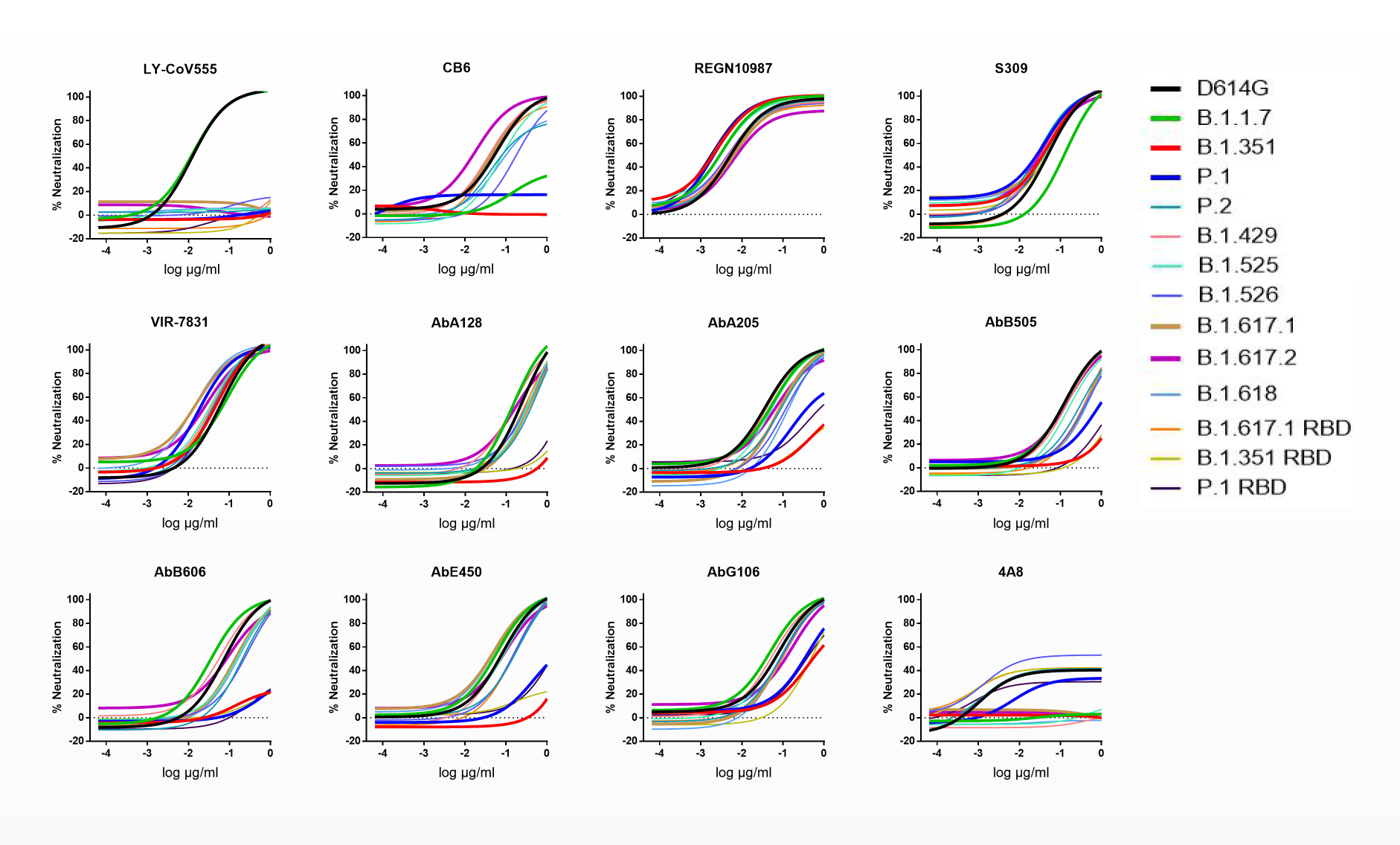

Supplement: Supplementary Figure 4 — Neutralization of SARS-CoV-2 variants by mAbs, related to Figures 4 . Pseudoviruses carrying the variant S proteins were tested against a series of dilutions of each mAb. Neutralization activity was defined as the percentage of decrease in luciferase activity compared to the virus control wells (virus + cells). [file Image_4.tif]

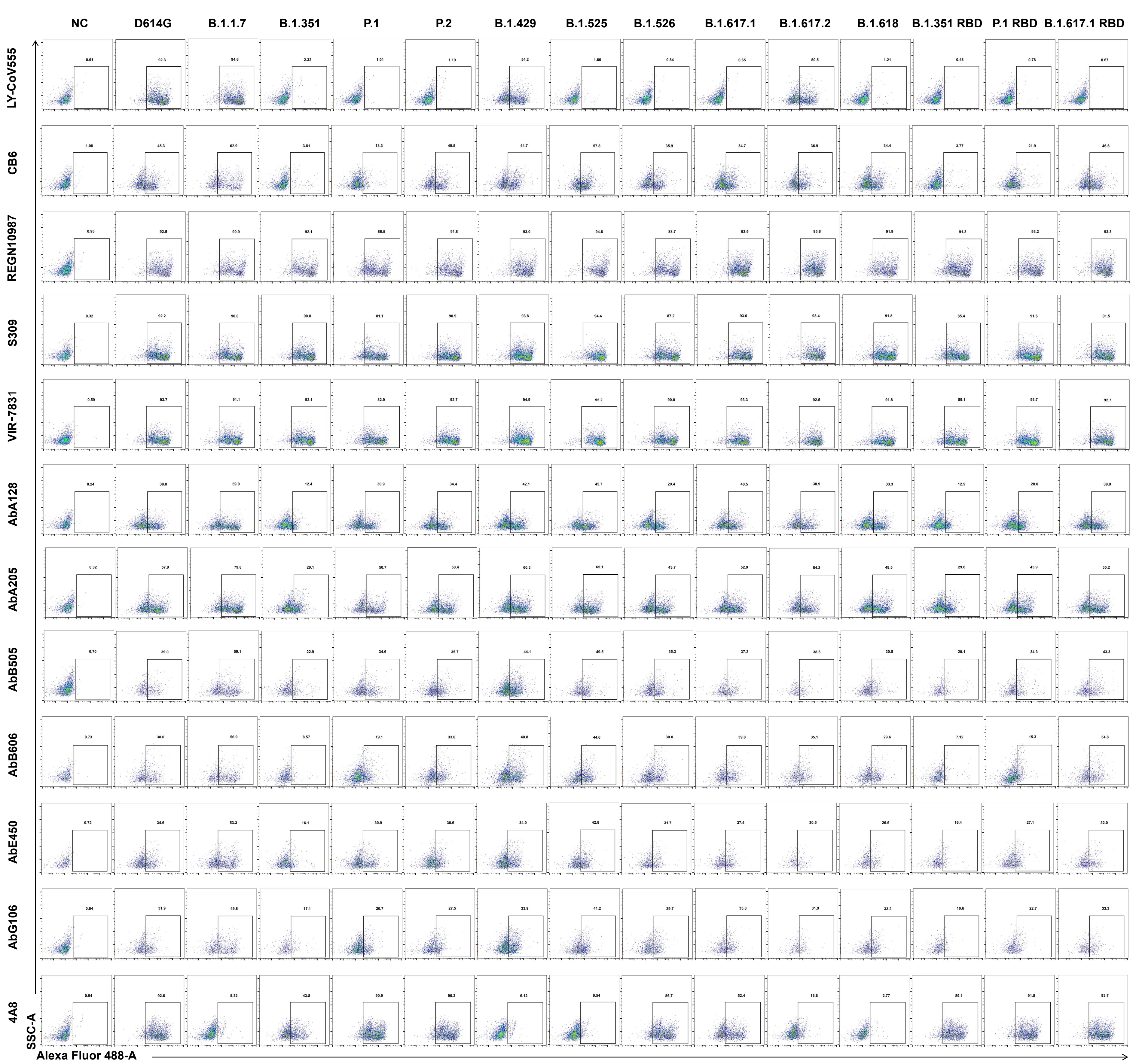

Supplement: Supplementary Figure 5 — Binding to cell surface expressed SARS-CoV-2 variants S proteins by neutralizing mAbs, related to Figures 4 . SARS-CoV-2 variants S proteins were expressed on the surface of 293T cells, incubated with anti-SARS-CoV-2 spike antibody and neutralizing mAbs, followed by staining with Alexa Flour 488-conjugated anti-human IgG Fc and PE-conjugated anti-mouse IgG antibodies, and analyzed by flow cytometry. Experiments were done once. NC is 293T cells with mock transfection. [file Image_5.pdf]

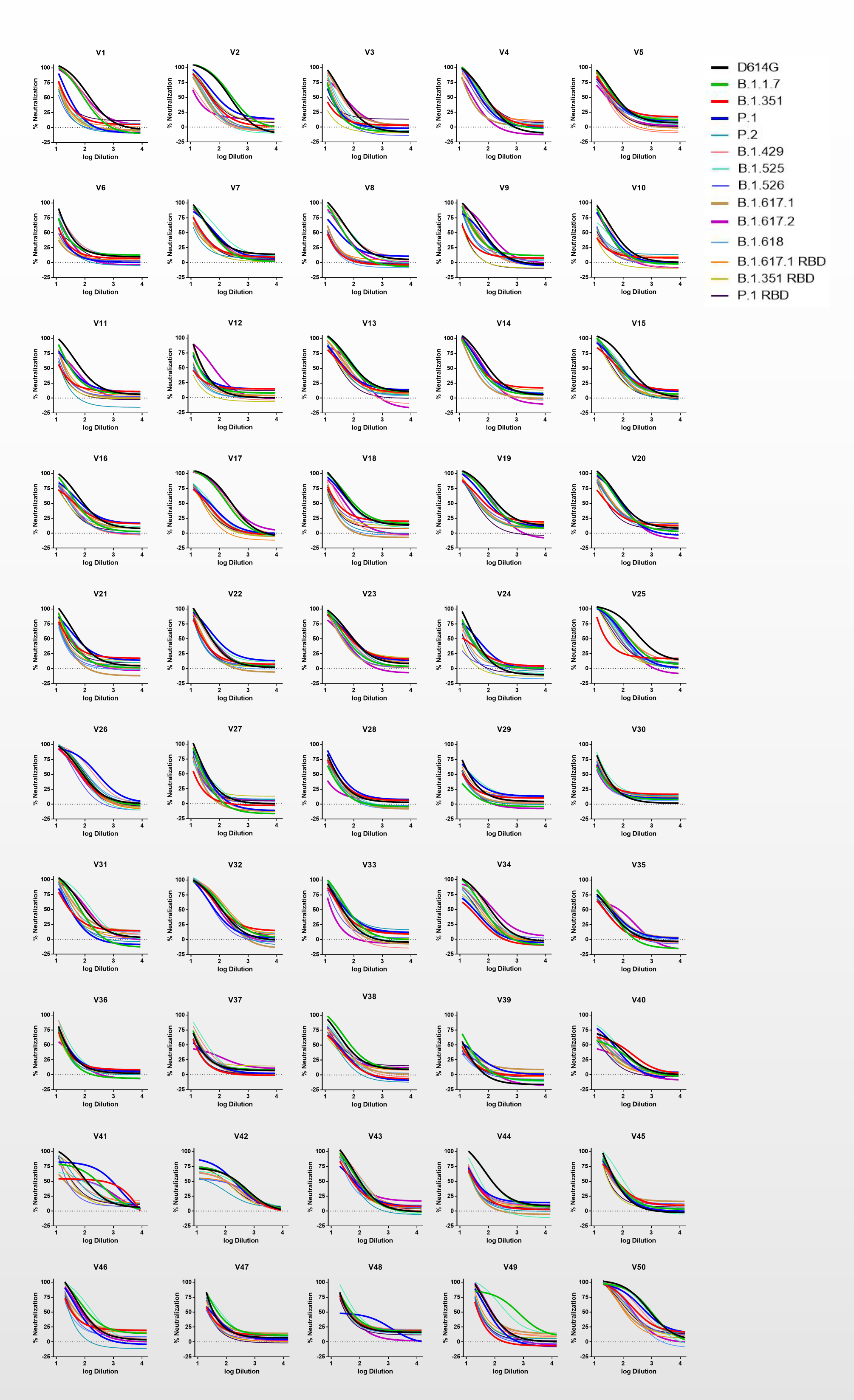

Supplement: Supplementary Figure 6 — Neutralization of SARS-CoV-2 variants by vaccine sera, related to Figures 5 . Pseudoviruses carrying the indicated variant S proteins were tested against serial dilutions of vaccine sera [CoronaVac vaccine (V1-V30) or BBIBP-CorV vaccine (V31-V50)]. Neutralization activity was defined as the percent reduction in luciferase activity relative to the virus control wells (virus + cells). [file Image_6.tif]

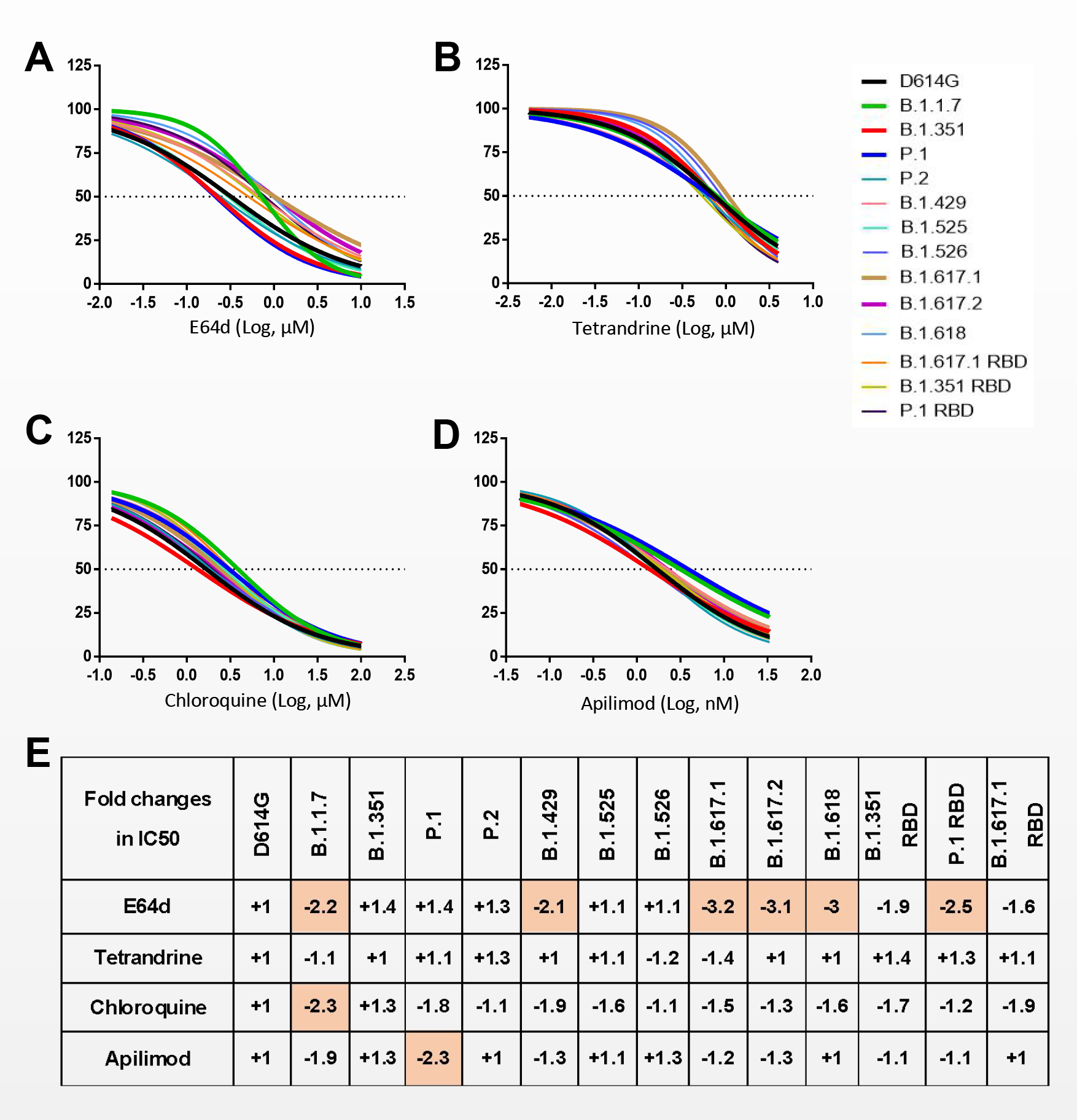

Supplement: Supplementary Figure 7 — SARS-CoV-2 S pseudovirus entry into 293T-hACE cells can be blocked with endocytosis and protease inhibitors, repeated experiments of Figures 6 . [file Image_7.tif]
